# Uncovering the Time-Frequency Relationship Between Google Trends and COVID-19 Vaccination Metrics: A Hybrid ARDL-Wavelet Coherence Model for Prediction

**DOI:** 10.7759/cureus.93193

**Published:** 2025-09-25

**Authors:** Zongjing Liang, Gongcheng Liang, Yun Kuang, Zhijie Li

**Affiliations:** 1 School of Economics and Management, Guangxi Normal University, Guilin, CHN; 2 Network and Educational Technology Center, Guilin Normal University, Guilin, CHN; 3 Library, Guilin Normal University, Guilin, CHN

**Keywords:** ardl model, covid-19 vaccine, frequency-domain analysis, google trends, information be-havior, time–frequency analysis, vaccine prediction, wavelet coherence

## Abstract

Background/Objectives: With the continuous evolution of global infectious diseases such as the coronavirus disease 2019 (COVID-19) pandemic, vaccination remains one of the most effective means of intervention. However, in the process of vaccination promotion, public vaccination behavior often lags behind policy deployment. Accurately predicting vaccination trends early has become one of the key problems in the current public health field. At present, the public's online search (such as Google Trends (Google LLC, Mountain View, CA)) has become an important predictor of vaccination intervention. However, existing studies have low prediction accuracy and a lack of understanding of the dynamic heterogeneity between Google Trends and vaccination behavior. This study aims to improve the prediction accuracy of vaccination using the autoregressive distributed lag model (ARDL model) and solve the dynamic heterogeneity problem using wavelet coherence analysis.

Methods: This paper first uses the ARDL model to study the long-term cointegration relationship between the search index (Google Trends) and the COVID-19 vaccination rate in the United States (including the number of vaccination doses per week, the number of people who receive at least one dose of vaccine per week, and the number of people who complete the full vaccination per week). Then, in order to reveal the time-varying characteristics of the predictor variables, the wavelet coherence method is introduced to study the dynamic interaction between Google Trends and vaccination behavior in the time-frequency domain. There are only a few such relevant studies in the existing literature on vaccination prediction. The data for this study covers December 2020 to May 2022. Vaccination data comes from the US CDC, and Google Trends data comes from keywords extracted from COVID-19 vaccines.

Results: The results of the time domain study show that Google Trends has a significant long-term cointegration relationship with the weekly vaccination rate. Because this paper uses an ARDL model with automatically selected optimal lag orders, the prediction accuracy of the search index for vaccination has been significantly improved. After comparing the prediction accuracy with existing similar studies, the root mean square error (RMSE) and mean absolute error (MAE) obtained in this paper are much smaller than those reported in existing studies. The results of the frequency domain wavelet coherence study show that in the early stage of rapid vaccine promotion (especially in early 2021), Google Trends has a strong correlation with vaccination behavior. From the end of 2021 to 2022, the coherence between GT and vaccination behavior gradually weakened.

Conclusions: The results show that Google Trends has good long-term prediction ability in the vaccine promotion stage, but its short-term prediction effect shows volatility with time and frequency. The ARDL-wavelet coherence analysis framework proposed in this paper provides a new research paradigm for evaluating the public's influence on vaccination. The research results emphasize the potential and importance of Google Trends in the construction of real-time public health response and early warning systems.

## Introduction

According to statistics from the World Health Organization and health monitoring departments of various countries, between March and May 2025, monitoring data from the Hong Kong Special Administrative Region of China, Singapore, the United Kingdom, France, Brazil, and Norway showed that the number of people infected with the new coronavirus showed an increasing trend [1-4]. From the outbreak of the new coronavirus in 2020 to the end of the public health emergency declared by the WHO in 2023, three years have passed. The coronavirus disease 2019 (COVID-19) virus, one of the coronaviruses, has once again become prevalent, which undoubtedly puts forward new requirements for the prevention and control of epidemics in countries around the world. It can be observed that almost all public health emergencies since this century are related to coronaviruses [1]. In order to effectively respond to the spread of the COVID-19 virus, vaccination is undoubtedly an important part of all prevention and control measures.

Vaccination has always been one of the powerful means for humans to prevent and control infectious diseases. In particular, the recent epidemics of coronavirus-related infectious diseases have undoubtedly increased the importance of vaccination in countries around the world. Since the outbreak of the COVID-19 pandemic, the key role of vaccination in controlling the spread of infectious diseases has become increasingly prominent [5]. Although large-scale vaccination programs have been rapidly implemented worldwide, the effectiveness of these programs depends not only on vaccine supply but also on accurate and timely predictions of vaccination trends [6]. Only by accurately predicting vaccination trends can public health departments formulate and optimize intervention strategies in a targeted manner, thereby effectively responding to sudden epidemic fluctuations and achieving vaccine equity.

Current status of vaccination prediction

The current status of vaccination prediction can be divided into two parts. The first part is the prediction method. Vaccination behavior is affected by many factors, and the prediction of vaccination needs to consider the role of various influencing variables. In the Internet era, researchers use Internet data to predict vaccination behavior in order to improve the accuracy and timeliness of prediction. The current vaccine prediction method mainly combines Internet big data for real-time prediction. The specific big data used includes search index, social media, hybrid data model method, etc. [7]. The application results of social media data used social media data to study vaccine hesitancy and vaccination behavior. The results show that there is a correlation between social media activities and vaccine hesitancy [8]. The research results of multi-source hybrid data prediction are to combine Internet big data with traditional clinical data to achieve hybrid data vaccine prediction [9].

The second part is the prediction tool. At present, for the time series data of vaccination rate, the prediction tool model can be summarized as follows: Using (ARIMA + linear regression) to predict vaccination rates in Africa, the prediction accuracy RMSE 0.0305) [10]. The CNN-LSTM model was used to predict global vaccination, with an average absolute percentage error of 3.1% [11]. Using logistic regression, random forest, and balanced random forest (BRF) for inoculation prediction, the accuracy rate is about 74% [12]. The ensemble learning method combining clinical and network data was used to predict vaccination rates, with an average RMSE of 4.7 [13]. The time series analysis method was used to predict BCG and OPV demand, with the average R-squared value of the model fitting coefficient R2=0.7175 [14]. The statistical model and machine learning algorithm were used to predict vaccination willingness, with an accuracy of about 85% for predicting individual vaccination willingness [15]. The ARIMA model was used to predict the trend of COVID-19 vaccination, with an average error index of about 0.6672 [16]. The search data was used to predict the trend of COVID-19 vaccination, with an average RMSE of 5.923 and an average MAE of 4.657 [17]. The predictors of confidence and willingness to vaccinate for COVID-19 and influenza in the United States were studied and compared, with an overall average standard deviation of 1.27 [18]. Error analysis of the mixed time series model in the prediction of COVID-19 vaccination, with RMSE and MAE of approximately 2.185 and 1.7275 [19]. 

The autoregression analysis lag model (ARDL model) is a time series statistical model with lagged variables. This model can be used as a regression model to analyze the dynamic relationship between variables in the short and long term. The theory was first developed by Pesaran et al. [20,21]. The ARDL model is suitable for time series data, especially when the variables are integrated at different orders, and can simultaneously estimate short-term effects and long-term cointegration relationships under small samples. This model has been used in economics, medicine, environmental science, and other fields [22-24]. However, no research results using the ARDL model for vaccine prediction have been published so far.

Research status review

Current vaccine prediction research methods mainly focus on search index(such as Google Trends), social media, or multi-source data hybrid methods, and have achieved corresponding research results. The research scope is limited to time domain analysis. Although many achievements have been made in the field of vaccine prediction, there are still the following issues that deserve further study: (1) The accuracy of real-time prediction needs to be improved. The prediction accuracy of existing research results is generally not very high due to various reasons, and prediction accuracy is very important for policy reference. Although the Centers for Disease Control and Prevention (CDC) releases statistics on vaccination in the United States every day, these data usually have a delay of 1-3 weeks [7]. Therefore, high-precision real-time prediction has important practical value. (2) There is a lack of research on the long-term effect of Google Trends on vaccination prediction. This effect analysis plays an important role in public health management, epidemic response efficiency, resource allocation optimization, and vaccine strategy formulation. (3) Current research lacks frequency domain research on vaccination (i.e., studying the characteristics and laws of Google Trends and vaccination behavior data in the frequency dimension). Frequency domain research can reveal the co-evolution relationship between information dissemination, public behavior, and vaccination rate at different frequencies (i.e., different time scales), which is particularly important for formulating real-time response policies.

Research objectives

Based on the shortcomings of current research, this paper proposes three research objectives: (1) Construct a new vaccination prediction model. The specific method is to construct a Google Trends comprehensive index of multiple keywords, and then use the ARDL model to implement a new vaccination prediction model, and improve the prediction accuracy by automatically setting the lag order of the variables. (2) Construct a long-term effect equation for vaccination prediction. The specific method is to solve the ARDL model and calculate the long-term cointegration effect equation between the search index and vaccination. (3) Conduct a wavelet coherence study on the search index and vaccination. By using a MATLAB program (The Mathworks, Inc., Natick, MA) to obtain a wavelet coherence graph between the two, the relationship between the search index and vaccination behavior data is studied from the frequency dimension.

The research results are expected to provide useful reference data for current and future responses to major infectious disease outbreaks.

## Materials and methods

The data for this study comes from two databases, of which the US COVID-19 vaccination data is taken from the Our World in Data (OWID) database, whose data comes from the US CDC [25]. The public attention data (i.e., Google Trends) is taken from the Google Trends website [26]. The research methods are the ARDL model and wavelet coherence analysis. The vaccination prediction dependent variable is weekly vaccination data (weekly vaccination doses, number of people who receive at least one dose of vaccine per week, number of people who complete the full vaccination per week), and the independent variable is Google Trends. Eviews 10 software (S&P Global, New York, NY) is used to calculate the ARDL model, and Matlab R2022, a software is used for wavelet coherence calculation.

Data

The data time range of the original data of US vaccination downloaded from the OWID database [25] is from December 13, 2020, to May 31, 2022. The vaccination data in the CDC database is daily data, while the search index within this time period is weekly data. Therefore, in order to unify the variable data dimension, the vaccination data is converted into weekly data in the actual calculation. The data variables include US COVID-19 weekly vaccinations, weekly people vaccinated, and Weekly people fully vaccinated, represented by Y1, Y2, and Y3, respectively. The Google Trends [26] data comprised search keywords such as "covid vaccine," "pfizer covid vaccine," "moderna covid vaccine," "johnson and johnson covid vaccine," "covid vaccine side effects," "covid vaccine safe," "covid vaccine effective," "covid vaccine appointment," "covid vaccine eligibility," "where to get covid vaccine."

 ARDL model

The ARDL model was first proposed by Pesaran et al. [27]. This model is an extension of the ordinary regression model. Its basic principle is to add a dynamic new variable relationship model with lagged variables on the basis of the static variables of the ordinary regression model. The long-term cointegration relationship between the number of vaccinations (Y) and the search index studied in this paper can be expressed by the equation below [28]. The optimal lag order of the ARDL model is determined by AIC. The long-term cointegration relationship between variables can be obtained through the ARDL model. Compared with the traditional cointegration model, this cointegration relationship has three advantages. The first is that the requirements for original data are broadened. The second is that the model is suitable for small sample data sets. The third is that the F value can be used to determine whether the cointegration relationship exists [28].

$Y_{i} = \alpha_{0} + \sum_{i=1}^{m} \alpha_{1,i} Y_{it-i} + \sum_{i=0}^{p} \alpha_{2,i} GT_{t-i} + \sum_{i=0}^{n} \alpha_{3,i} EC_{t-i}$

Yi (i=1,2,3) in the formula represents three types of vaccination variables. Among them, Y1 corresponding to weekly vaccinations, refers to the total number of vaccine doses administered each week; Y2, corresponding to weekly people vaccinated, refers to the number of individuals who received at least one dose of a COVID-19 vaccine each week; and Y3, corresponding to weekly people fully vaccinated, refers to the number of individuals who became fully vaccinated each week. α_0_ is a constant term. ∑m_ i=1_ α_1,i_ Yi_t-i_ Yi is the lag term that represents the influence of the dependent variable at the previous time. ∑p _i=0_ α_2,i_ GT_t-i_ is the lagged term of Google Trends, reflecting the impact of Google Trends on the dependent variable. ∑n_ i_=0 α_3_,_i_ EC_t-i_ is the error correction term (EC), which represents the degree of deviation from the long-term equilibrium of the system.

Wavelet coherence

Wavelet coherence is used to study the relationship between two time series in the time-frequency space. Wavelet coherence (WTC) can be regarded as the local correlation between two continuous wavelet transforms. In this way, local phase locking behavior can be discovered [29]. In specific practical operations and applications, the value range of wavelet coherence is Greater than or equal to 0 and less than or equal to 1. The closer the value is to 1, the stronger the correlation between the two signals at this time and frequency. The wavelet coherence diagram can be visualized, where the horizontal axis represents time, the vertical axis represents frequency, and the color depth represents the coherence size [29]. WTC analysis can measure the dynamic linkage relationship and lead-lag relationship between time series at different frequencies [30].

## Results

Vaccination data

**Figure 1:**
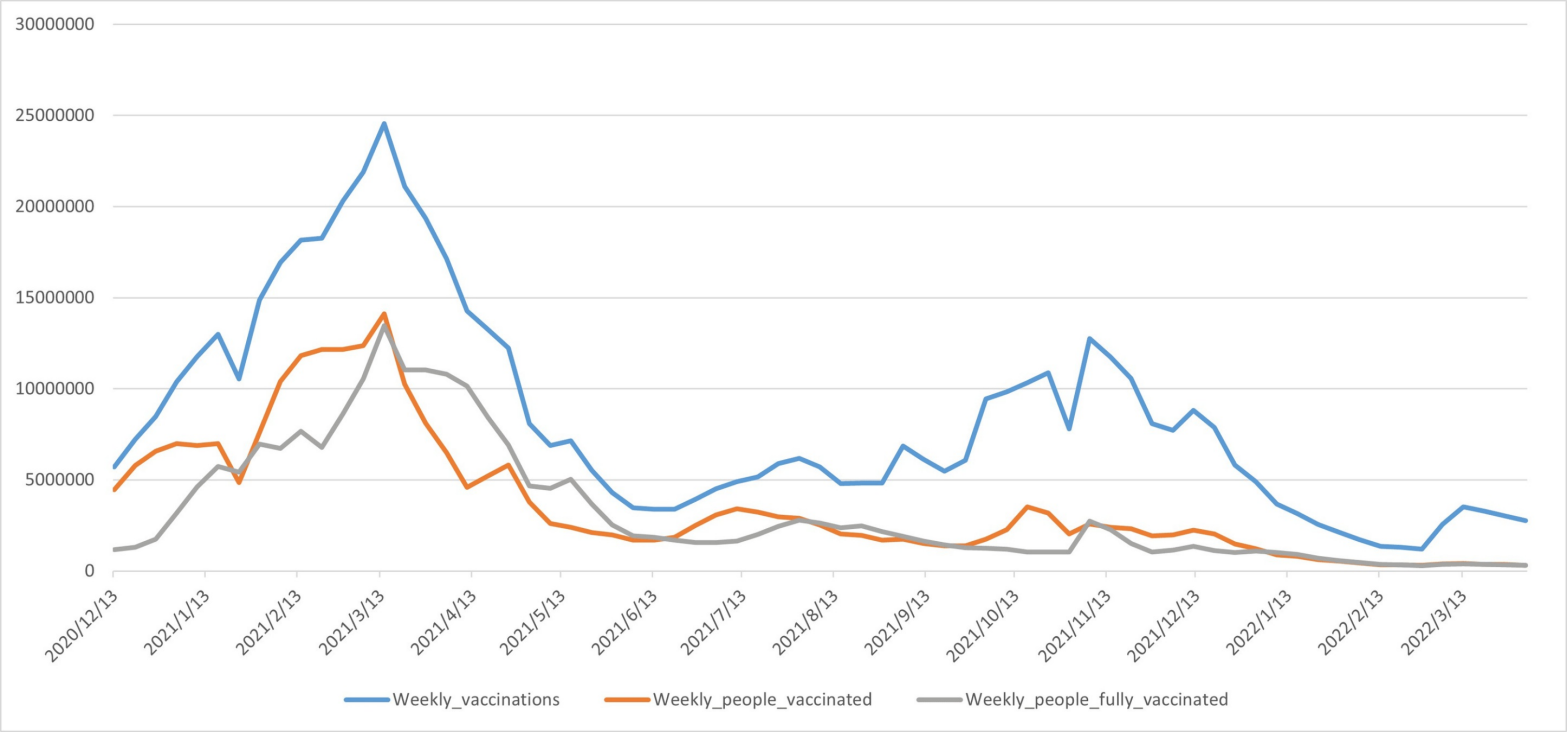
Line charts of three vaccination data

The original data on US vaccinations were downloaded from the OWID database [27]. The data range is from December 13, 2020, to May 31, 2022. The data variables include US COVID-19 weekly vaccinations, weekly people vaccinated, and weekly people fully vaccinated, represented by Y1, Y2, and Y3, respectively. The specific data are shown in Figure 1, which shows that vaccination data experienced a peak cycle from December 2020 to June 2021, reaching its first peak around March 2021. From June 2021 to February 2022, vaccination data experienced a second peak cycle, reaching its peak in November 2021. The figure also reveals a gradual downward trend in vaccination data. Table 1 below shows a statistical description of the raw data for the three vaccinations.

**Table 1:** Statistical description table of three vaccination data This table presents the descriptive statistics of the weekly vaccination used in this study, including COVID-19 vaccination metrics in the United States from December 13, 2020 to May 31, 2022. Variables include: weekly vaccinations (number of total weekly COVID-19 vaccine doses administered), weekly people vaccinated (number of people receiving at least one dose), and weekly people fully vaccinated (number of people completing the full vaccine regimen). Statistical indicators shown are: mean, median, maximum, minimum, standard deviation (Std. Dev.), skewness, kurtosis, Jarque-Bera test value (for normality), and its associated probability. Sum and sum of squared deviations (Sum Sq. Dev.) are also included.

Indicators	Weekly vaccinations	Weekly people vaccinated	Weekly people fully vaccinated
Mean	8345524	3641845	3208538
Median	6849628	2328997	1750076
Maximum	24556882	14125060	13477122
Minimum	1190791	320407	282502
Std. Dev.	5615500	3435504	3267382
Skewness	1.032597	1.449899	1.441426
Kurtosis	3.318536	4.256896	4.057632
Jarque-Bera	12.55366	28.71727	27.1096
Probability	0.001879	0.000001	0.000001
Sum	5.76E+08	2.51E+08	2.21E+08
Sum Sq. Dev.	2.14E+15	8.03E+14	7.26E+14

This paper used the ARDL model for vaccine prediction analysis. According to the modeling requirements of the ARDL model, the original data must be tested for stationarity. This paper uses the ADF method for the stationarity test. The test results are shown in Table 2, which shows the ADF unit root test results of each variable. As can be seen from the table, the original data series of the four variables are not stable, but they become stable series after the first-order difference. The test reports the ADF test statistic and the corresponding critical values at the 1%, 5%, and 10% significance levels. A series is considered stationary if the test statistic is less than the critical value at the desired significance level. This shows that the three variables are all first-order single integration I(1). According to the modeling requirements of the ARDL model, the above three variables can be subjected to an ARDL cointegration analysis.

**Table 2:** Augmented Dickey-Fuller (ADF) test results for assessing the stationarity of the original data series. The table presents ADF unit root test statistics for each variable. The test includes critical values at the 1%, 5%, and 10% significance levels (i.e., p < 0.01, p < 0.05, p < 0.10). A series is considered stationary if the test statistic is less than the critical value at the corresponding significance level. All variables become stationary after first differencing and are integrated of order one, I(1). The test was performed using EViews 10.

Variable Description	Variable Code	t-Statistic Level	First difference	Test critical values: 1% level	Single integral form
Weekly vaccinations	Y1	-1.046	-6.896	-3.531592	I(1)
Weekly people vaccinated	Y2	-0.994	-6.239	-3.531592	I(1)
Weekly people fully vaccinated	Y3	-0.999	-6.315	-3.531592	I(1)

Google Trends data

To comprehensively collect Google Trends data related to COVID-19 vaccination and better reflect the diversity of public search behavior, this article selected 10 keywords closely related to COVID-19 vaccination. Principal component analysis was then used to integrate these 10 sets of search data into a comprehensive search index. The definitions and rationale for the selected keywords are shown in Table 3, which provides a definition table of 10 Google Trends search keywords. This table explains the principles for keyword selection. These 10 keywords were selected based on multiple considerations, including general keywords, brand keywords, information acquisition and safety-related keywords, and vaccination behavior and access keywords. By collecting Google Trends data for these four keyword categories, we can extract comprehensive personal data on vaccination behavior, providing objective data for studying the correlation between individual intentions and actual behavior.

**Table 3:** 10 Google Trends search keyword definitions

No.	Search Keyword	Variable Name	Description
1	Covid vaccine	GT1	General keyword. The most fundamental and core search term.
2	Pfizer Covid vaccine	GT2	Brand keyword. Pfizer is one of the main vaccine suppliers in the United States.
3	Moderna Covid vaccine	GT3	Brand keyword. Moderna is one of the main vaccine suppliers in the United States.
4	Johnson and Johnson Covid vaccine	GT4	Brand keyword. Johnson & Johnson had significant attention from 2021 to 2024.
5	Covid vaccine side effects	GT5	Information and safety-related keyword. This keyword focuses on vaccine side effects.
6	Covid vaccine safe	GT6	Information and safety-related keyword. This keyword focuses on vaccine safety.
7	Covid vaccine effective	GT7	Information and safety-related keyword. This keyword focuses on vaccine effectiveness.
8	Covid vaccine appointment	GT8	Behavior and access-related keyword. This keyword reflects the intention of making a vaccination appointment.
9	Covid vaccine eligibility	GT9	Behavior and access-related keyword. This keyword reflects the intention regarding vaccination eligibility.
10	Where to get Covid vaccine	GT10	Behavior and access-related keyword. This keyword reflects the intention regarding vaccination locations.

**Figure 2:**
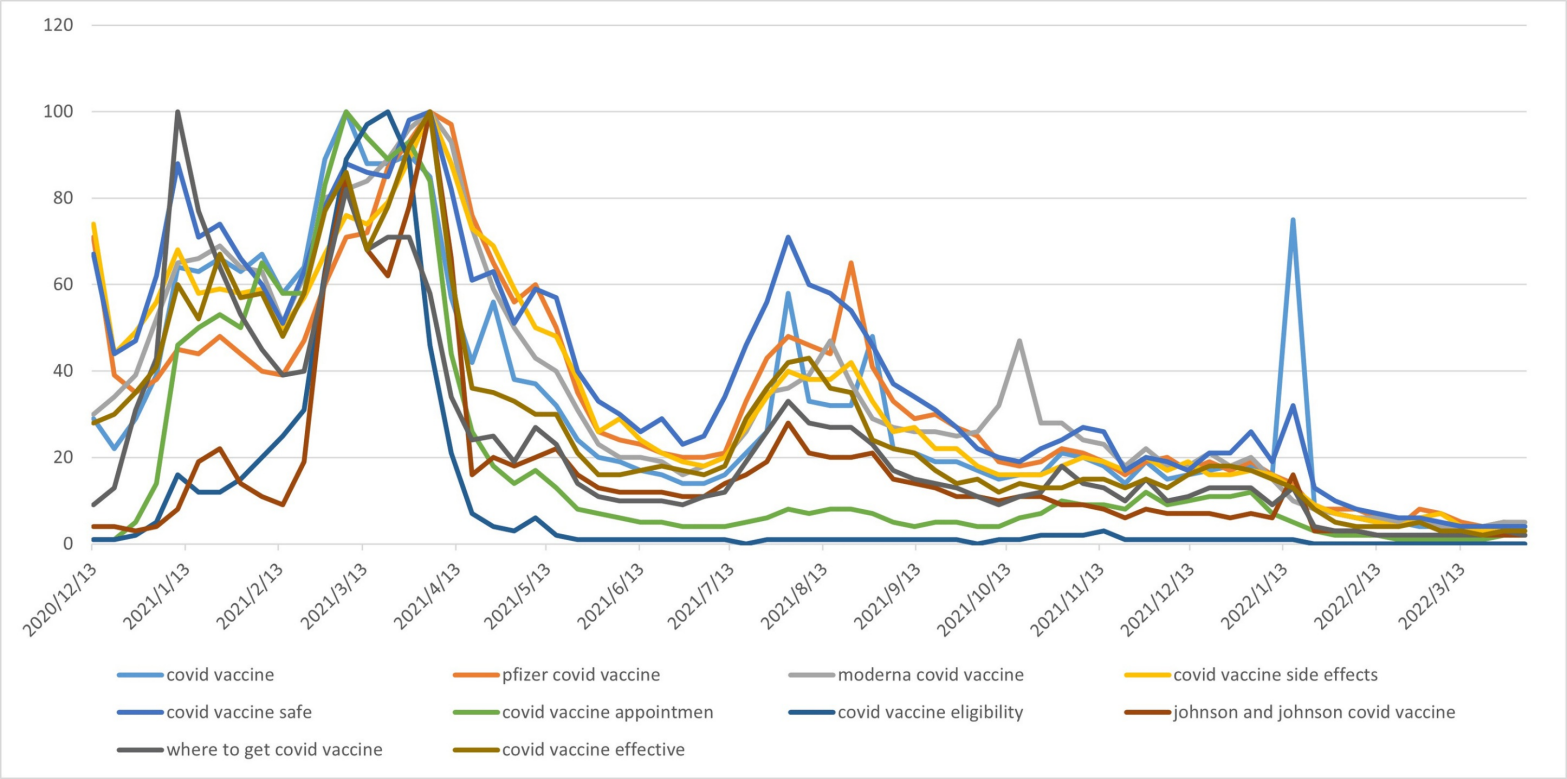
Google Trends data line chart for 10 search keywords In the figure, the blue curve represents "covid vaccine", the orange curve represents "Pfizer covid vaccine", the gray curve represents "moderna covid vaccine", the yellow curve represents "covid vaccine side effects", the light blue curve represents "covid vaccine safe", the brown curve represents "covid vaccine appointment", the light gray curve represents "where to get covid vaccine", the green curve represents "covid vaccine effective", the light cyan curve represents "covid vaccine eligibility", and the dark brown curve represents "Johnson and Johnson covid vaccine".

The Google Trends data for the 10 search keywords is shown in Figure 2. As can be seen from the figure, the 10 data line graphs show a relatively consistent evolutionary trend. Around April 2021, there was a peak, followed by a decline, then an increase. Around August 2021, the data reached a second peak, followed by a gradual decline. Table 4 shows the Google Trends data statistics for the 10 search keywords.

**Table 4:** Google Trends data statistical description table of 10 search keywords

Parameter	GT1	GT2	GT3	GT4	GT5	GT6	GT7	GT8	GT9	GT10
Mean	32.23188	34.3913	35.23188	17.34783	34.50725	40.34783	28.95652	19.4058	10.21739	23.5942
Median	21	27	27	11	26	33	18	7	1	14
Maximum	100	100	100	100	100	100	100	100	100	100
Minimum	3	4	4	2	3	4	2	1	0	2
Std. Dev.	25.87364	24.08699	25.5942	20.74412	25.03681	26.34322	23.62603	27.11865	23.49084	22.48811
Skewness	1.038669	0.937442	0.8902	2.420079	0.728195	0.468709	1.19324	1.773537	2.88061	1.504812
Kurtosis	2.958194	3.257779	2.886558	8.232327	2.485094	2.186959	3.706187	4.82141	10.23217	4.549247
Jarque-Bera	12.41161	10.29722	9.150239	146.0625	6.860317	4.426891	17.80772	45.7104	245.8007	32.94175
Probability	0.002018	0.005807	0.010305	0	0.032382	0.109323	0.000136	0	0	0
Sum	2224	2373	2431	1197	2381	2784	1998	1339	705	1628
Sum Sq. Dev.	45522.29	39452.43	44544.29	29261.65	42625.25	47189.65	37956.87	50008.64	37523.74	34388.64

Table 4 shows a statistical descriptive table of Google Trends data for 10 search keywords. As can be seen from the table, the search data for the top 10 keywords exhibits significant variation in search interest, with peaks concentrated. Most keywords experience short bursts of search activity during specific time periods. The figure also reveals significant volatility, likely due to the influence of external events, news, and other factors on public interest. To analyze this data using the ARDL model, it is necessary to test its stationarity using the ADF indicator. The results are shown in Table 5, which shows the ADF test results for the stationarity of Google Trends data for 10 search keywords. As can be seen from the table, nine of the 10 search data sets were originally non-stationary data series. After first-order differencing, they all became stationary data series at a 1% error level, achieving first-order integration. The search data series for the keyword "covid vaccine eligibility" was originally stationary, that is, zero-order integrated. According to the ARDL model's modeling requirements, as long as the original data series is first-order or zero-order integrated, the model's requirements are met. 

**Table 5:** ADF test of the stationarity of Google Trends data for 10 search keywords The table presents ADF unit root test statistics for each variable. The test includes critical values at the 1%, 5%, and 10% significance levels (i.e., p < 0.01, p < 0.05, p < 0.10). A series is considered stationary if the test statistic is less than the critical value at the corresponding significance level. The test was performed using EViews 10.

Search keywords	Variable name	Original data series; t-statistic level	t-statistic level after one-price difference	t-statistic at the 1% significance level	Single integral form
Covid vaccine	GT1	-1.58427	-11.0364	-3.531592	I(1)
Pfizer covid vaccine	GT2	-1.278466	-7.376449	-3.531592	I(1)
Moderna covid vaccine	GT3	-1.354762	-5.680838	-3.531592	I(1)
Johnson and Johnson covid vaccine	GT4	-1.87065	-7.926223	-3.533204	I(1)
Covid vaccine side effects	GT5	-0.775374	-8.098351	-3.531592	I(1)
Covid vaccine safe	GT6	-1.271378	-7.6424	-3.531592	I(1)
Covid vaccine effective	GT7	-1.396256	-5.822437	-3.533204	I(1)
Covid vaccine appointment	GT8	-2.051756	-3.494267	-3.534868	I(1)
Covid vaccine eligibility	GT9	-3.556117	-	-3.531592	I(0)
Where to get covid vaccine	GT10	-2.150295	-7.127695	-3.531592	I(1)

**Figure 3:**
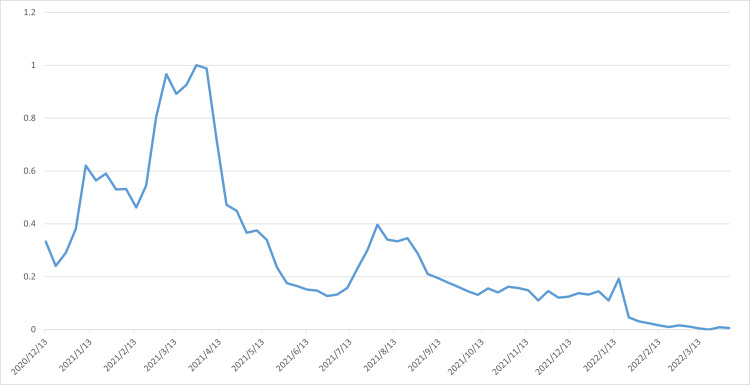
Google Trends (GT) comprehensive score chart obtained by principal component analysis of GT data for 10 search keywords (GT data has been normalized to 0-1).

For ease of calculation, this article performs an index conversion on the 10 search data sets. This conversion method uses principal component analysis. The following combined search index is obtained, as shown in Figure 3, which shows the Google Trends composite score for 10 search keywords, derived through principal component analysis. This figure converts the 10 search data sets into a new variable score, which reflects the common variation characteristics of these 10 search groups. Combining this data with the vaccination data provides the raw data needed for this study. This paper uses this composite score as the search index for vaccination, denoted by the variable Google Trends.

**Figure 4:**
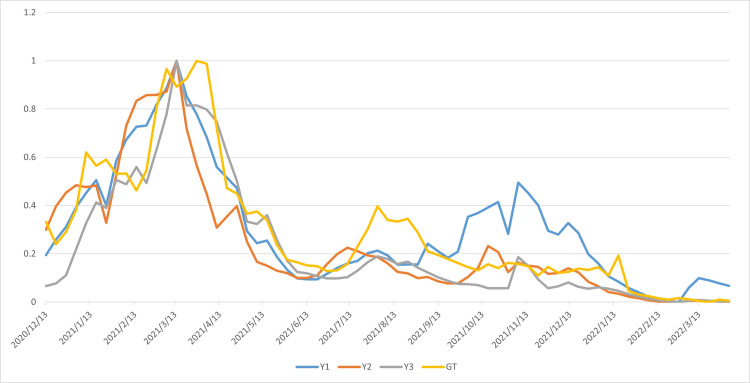
Time series diagram of Y1, Y2, Y3, and Google Trends data after 0-1 standardization The figure shows the weekly trends of Y1 (weekly vaccinations), Y2 (weekly people vaccinated), Y3 (weekly people fully vaccinated), and Google Trends. All variables were rescaled to a common range of 0–1 to facilitate visual comparison of dynamic trends over time. The y-axis represents the normalized values, and the x-axis represents time (in weeks).

Because the vaccination data and search data have different dimensions and significant numerical differences, to facilitate analysis of correlations between the variables, this paper normalizes the vaccination data and composite search data scores to a 0-1 scale, with GT representing the search index. The results of this normalization are shown in Figure 4.

In the figure, Y1, Y2, Y3, and GT represent weekly vaccinations, weekly people vaccinated, weekly people fully vaccinated, and Google Trends, respectively. This figure also shows the general trend of change in the four variables. From December 2020 to June 2021, there was a first peak and then a decline, with the first peak reaching around March 2021. After that, the values of all four variables declined. The four data series show that they have the same trend of change, indicating that the search index may have predictive properties. Subsequently, each variable showed a downward trend, indicating that vaccination is gradually entering its final stage, and public attention is also showing a downward trend. Overall, GT and Y1, Y2, and Y3, that is, vaccination behavior, have a certain leading and predictive nature.

Vaccination prediction

This paper uses the ARDL model for vaccination prediction. The dependent variables of the prediction model are Y1, Y2, and Y3, and the independent variable is GT. The calculation tool EViews 10 software was used. The following describes the steps of calculating the three dependent variables of the ARDL model.

 ARDL Calculation Results

The calculation results of Y1, Y2, and Y3 ARDL models are shown in Tables 6-8.

**Table 6:** Y1 ARDL estimation results In the table, ARDL(7,7) represents the ARDL structure type of Y1, indicating that in the Y1ARDL model, Y has a 7th-order lag term and X has a 7th-order lag term.

Variable	Coefficient	Std. Error	t-Statistic	Prob.*	95% CI Low	95% CI High
Y1(-1)	0.762914	0.130629	5.840318	0	0.499972	1.025856
Y1(-2)	0.086703	0.168349	0.515021	0.609	-0.252165	0.425572
Y1(-3)	0.135435	0.171196	0.791113	0.4329	-0.209164	0.480034
Y1(-4)	0.13016	0.170859	0.761795	0.4501	-0.213762	0.474081
Y1(-5)	0.260044	0.17386	1.49571	0.1416	-0.089918	0.610006
Y1(-6)	-0.210692	0.170745	-1.233959	0.2235	-0.554383	0.132999
Y1(-7)	-0.478294	0.148037	-3.230905	0.0023	-0.776278	-0.180311
GT	0.138275	0.140941	0.981085	0.3317	-0.145425	0.421975
GT(-1)	-0.062695	0.204812	-0.306108	0.7609	-0.474959	0.34957
GT(-2)	-0.241721	0.204916	-1.17961	0.2442	-0.654196	0.170754
GT(-3)	0.183837	0.18852	0.975157	0.3346	-0.195635	0.563309
GT(-4)	-0.154773	0.184857	-0.837259	0.4068	-0.526871	0.217324
GT(-5)	0.151014	0.181146	0.833655	0.4088	-0.213615	0.515642
GT(-6)	-0.069167	0.172754	-0.400379	0.6907	-0.416902	0.278568
GT(-7)	0.237446	0.109867	2.16121	0.0359	0.016295	0.458598
C	0.040139	0.014029	2.86106	0.0063	0.011899	0.068379
ARDL structure	ARDL(7, 7)					
R-squared	0.961325					

**Table 7:** Y2 ARDL estimation results In the table, ARDL(7, 11) represents the ARDL structure type of Y2, indicating that in the Y2 ARDL model, Y has a 7th-order lag term and X has a 11th-order lag term.

Variable	Coefficient	Std. Error	t-Statistic	Prob.*	95% CI Low	95% CI High
Y2(-1)	1.066341	0.135335	7.879252	0	0.792369	1.340314
Y2(-2)	-0.303423	0.20845	-1.455619	0.1537	-0.725407	0.118561
Y2(-3)	0.379419	0.222253	1.707144	0.096	-0.07051	0.829347
Y2(-4)	-0.388195	0.221854	-1.749777	0.0882	-0.837315	0.060925
Y2(-5)	0.498714	0.20345	2.451288	0.0189	0.086852	0.910577
Y2(-6)	-0.084283	0.210233	-0.400901	0.6907	-0.509877	0.341312
Y2(-7)	-0.410299	0.186859	-2.19577	0.0343	-0.788576	-0.032023
GT	-0.026716	0.188718	-0.141567	0.8882	-0.408755	0.355323
GT(-1)	0.202023	0.213553	0.946011	0.3501	-0.230292	0.634338
GT(-2)	-0.382799	0.215411	-1.777067	0.0836	-0.818875	0.053277
GT(-3)	0.00474	0.205424	0.023074	0.9817	-0.41112	0.4206
GT(-4)	0.095739	0.201504	0.475124	0.6374	-0.312183	0.503662
GT(-5)	0.129501	0.164619	0.78667	0.4364	-0.203753	0.462756
GT(-6)	-0.009442	0.163677	-0.057688	0.9543	-0.34079	0.321905
GT(-7)	0.356489	0.151797	2.348459	0.0242	0.049192	0.663785
GT(-8)	-0.266918	0.159931	-1.668959	0.1033	-0.590682	0.056845
GT(-9)	0.12057	0.165901	0.726758	0.4718	-0.215279	0.456419
GT(-10)	-0.328494	0.152897	-2.148467	0.0381	-0.638017	-0.01897
GT(-11)	0.250168	0.086636	2.887573	0.0064	0.074782	0.425553
C	-0.001118	0.012139	-0.092129	0.9271	-0.025692	0.023455
ARDL structure	ARDL(7, 11)					
R-squared	0.97114					

**Table 8:** Y3 ARDL estimation results In the table, ARDL(7, 7) represents the ARDL structure type of Y3, indicating that in the Y3 ARDL model, Y has a 7th-order lag term and X has a 7th-order lag term.

Variable	Coefficient	Std. Error	t-Statistic	Prob.*	95% CI Low	95% CI High
Y3(-1)	0.6458	0.127252	5.074952	0	0.3895	0.902099
Y3(-2)	0.083842	0.156942	0.534223	0.5958	-0.232256	0.39994
Y3(-3)	0.18052	0.171874	1.050305	0.2992	-0.165652	0.526692
Y3(-4)	0.181395	0.155235	1.168515	0.2488	-0.131265	0.494054
Y3(-5)	-0.079542	0.158191	-0.502819	0.6175	-0.398155	0.239072
Y3(-6)	0.126311	0.152178	0.830019	0.4109	-0.180191	0.432813
Y3(-7)	-0.392431	0.105994	-3.702395	0.0006	-0.605914	-0.178949
GT	0.121898	0.102195	1.192797	0.2392	-0.083933	0.32773
GT(-1)	0.342062	0.143757	2.379446	0.0216	0.052521	0.631603
GT(-2)	-0.22877	0.147095	-1.555251	0.1269	-0.525034	0.067495
GT(-3)	-0.122567	0.141693	-0.86502	0.3916	-0.407951	0.162817
GT(-4)	-0.246736	0.127373	-1.937113	0.059	-0.503279	0.009807
GT(-5)	0.119416	0.132293	0.902666	0.3715	-0.147035	0.385867
GT(-6)	-0.138813	0.132138	-1.050517	0.2991	-0.404952	0.127326
GT(-7)	0.277523	0.104861	2.64657	0.0112	0.066321	0.488725
C	0.06381	0.042009	1.518955	0.1358	-0.020801	0.14842
@TREND	-0.001184	0.000622	-1.905258	0.0631	-0.002436	6.77E-05
ARDL structure	ARDL(7, 7)					
R-squared	0.983845					

The estimation results of the three ARDL models (Y1, Y2, and Y3) are analyzed below.

Y1 Model (ARDL(7,7)): The model estimation results are shown in Table 6. In this model, the coefficients of the following variables are significant at the 5% significance level. Among them, the coefficient of Y1(-1) is 0.762914, p=0.000, indicating that the variable has a positive and significant effect and has strong self-sustaining properties. The coefficient of Y1(-7) is -0.478294, p=0.0023, indicating that it has a negative effect. The coefficient of the variable GT(-7) is 0.237446, p=0.0359, proving that it has a significant positive effect, which means that when GT(-7) increases by one unit, Y1 increases by 0.237446 units. In summary, Y1 shows positive inertia in the short term (1 period lag) and a significant negative correlation in the long term (7 periods). The impact of GT on Y1 has a significant medium- to long-term (7-period lag) effect.

Y2 Model (ARDL(7,11)): The model estimation results are shown in Table 7. In this model, the coefficients of the following variables are significant at the 5% significance level. Among them, the coefficients of Y2(-1), Y2(-5), and Y2(-7) are significant. The coefficients of the search data variables GT(-7), GT(-10), and GT(-11) are significant, among which GT(-7) and GT(-11) are positively significant, and GT(-10) is negatively significant. The coefficients for GT(-7), GT(-10), and GT(-11) are 0.356489, -0.328494, and 0.250168, respectively. This means that for every one-unit increase in GT(-7) and GT(-11), the corresponding vaccination variable Y2 increases by 0.356489 and 0.250168 units, respectively. However, for every one-unit increase in GT(-10), the corresponding vaccination variable Y2 decreases by 0.328494 units.

Y3 Model (ARDL(7,7)): The model estimation results are shown in Table 8. In this model, the coefficients of the following variables are significant at the 5% significance level. Among them, Y3(-1), GT(-1), and GT(-7) are positively significant, while the coefficient of Y3(-7) is negatively significant, indicating that Y3 is highly sensitive to both its own short-term and medium- to long-term lags. This also demonstrates that GT has both a direct short-term impact and a medium-term impact. The coefficients of GT(-1) and GT(-7) are 0.342062 and 0.277523, respectively, indicating that a one-unit increase in GT(-1) and GT(-7) leads to an increase in the corresponding vaccination variable Y3 by 0.342062 and 0.277523 units, respectively.

Overall Performance of the Three ARDL Models

The R² values for Y1, Y2, and Y3 are high, respectively: Y1 = 0.9613, Y2 = 0.9711, and Y3 = 0.9838. The average R² value for the three models is 0.97, indicating that the three models fit well. ARDL structure: Y1 (7,7), Y2 (7,11), Y3 (7,7), indicating that changes in the dependent variables of each model are influenced by their respective prior values. Regarding the search index GT (Google Trends), only GT(-7) in model Y1 is significant (0.2374, positive), indicating a significant delay in its response to search behavior. In model Y2, both GT(-7) (0.3565) and GT(-11) (0.2502) are significantly positive, demonstrating a long-lasting lag in its response to public concern. In model Y3, both GT(-1) (0.3421) and GT(-7) (0.2775) are significantly positive, indicating that they are both immediately affected by search behavior and reflected again over time.

ARDL boundary detection

Bounds Test Results

Following the ARDL model application steps, after calculating the regression equation, a bounds test is performed on the equation (equations 1 to 3 in Table 3 above) to verify whether a cointegration relationship truly exists between the variables. This test involves comparing the calculated F-statistics under the null hypothesis of no cointegration to determine whether the equation passes the bounds test. 

The ARDL model F-statistics for Y1, Y2, and Y3 were calculated to be F = 6.64, 4.99, and 4.97, respectively. Comparison with the Pesaran bounds test critical value table confirms whether the ARDL equations developed in this paper pass the bounds test. Table 9 shows the Pesaran bounds test critical value table.

**Table 9:** Pesaran boundary detection test critical value table This table presents the lower and upper bound critical values (I(0) and I(1)) at the 10%, 5%, 2.5% and 1% significance levels for the ARDL bounds test for cointegration. These critical values are used to determine whether a long-run cointegration relationship exists. Cointegration is indicated when the calculated F-statistic exceeds the upper bound value at the corresponding significance level. All F-values in this table are statistically significant.

Significance level	I(0) lower bound	I(1) upper bound	F-value judgment results
10%	3.02	3.51	significant
5%	3.62	4.16	significant
2.50%	4.18	4.79	significant
1%	4.94	5.58	significant

Because the F-statistic values of the ARDL model of Y1, Y2, and Y3A are F = 6.64, 4.99, and 4.97, respectively, these three values are significantly higher than the upper bound of I(1) at any significance level, the null hypothesis is rejected, indicating that there is a significant long-term cointegration relationship between Y1, Y2, Y3, and GT. The three long-term cointegration equations are:

$EC_{t} = Y_{1t} - \left( 0.5808 \cdot GT_{t} + 0.1279 \right)$

$EC_{t} = Y_{2t} - \left( 0.5993 \cdot GT_{t} - 0.0046 \right)$

$EC_{t} = Y_{3t} - \left( 0.4880 \cdot GT_{t} - 0.0047 \cdot Trend_{t} \right)$

where ECt is the error correction term. ARDL bounds test results indicate that the Google Trends indicator has a long-term positive impact on US vaccination data (Y1, Y2, and Y3). Combined with the long-term equation estimate, the long-term coefficient of GT for Y1 is 0.5808 (p < 0.001), indicating that for every one-unit increase in GT, Y1 will increase by approximately 0.5808 units in the long term. The coefficient of GT in the long-term equation for Y2 is 0.5993 (p < 0.001), highly significant, indicating that for every one-unit increase in GT, Y2 will increase by 0.5993 units. This conclusion further confirms the effectiveness of GT as a long-term predictor. The long-term coefficient for Y3 is significant, with a long-term impact coefficient of GT of 0.4880 (p < 0.001), meaning that for every one-unit increase in GT, Y3 will increase by approximately 0.4880 units in the long term.

Model stationarity test

After estimating the model, a diagnostic analysis was also performed, including a stationary test of the model. The stationary test of the model was mainly conducted by judging the cumulative sum (CUSUM) and cumulative sum of squares (CUSUMSQ) test to assess the stability of the estimated parameters. According to the graphs of the CUSUM and cumulative sum of squares of each model, it was confirmed that the estimated parameters of all three models were stable within the time range considered. The CUSUM and CUSUMSQ graphs of the empirical results show the stability of the long-term coefficients and short-term movements of the ARDL model. The results of the CUSUM and CUSUM SQ of the empirical results also show that the three models in this paper are stable at the 5% significance level.

ARDL prediction

The vaccination prediction method studied in this article utilizes both in-sample and out-of-sample forecasting methods. The in-sample forecast uses the entire data set, while the out-of-sample forecast uses data from December 13, 2020, to February 13, 2022, as the modeling data, and data from February 20, 2022, to April 3, 2022, as the prediction test data. The forecast error metrics used are root mean square error (RMSE) and mean absolute error (MAE). EViews 10 was used to calculate the in-sample and out-of-sample forecast error tables.

**Table 10:** Vaccination prediction error table In the table, RMSE and MAE represent the root mean square error and mean absolute error respectively.

Dependent variable	Y1		Y2		Y3	
Forecast Type	In-sample forecast error	Out-of-sample forecast error	In-sample forecast error	Out-of-sample forecast error	In-sample forecast error	Out-of-sample forecast error
RMSE	0.049	0.068	0.037	0.039	0.032	0.044
MAE	0.040	0.048	0.030	0.036	0.025	0.036

From the vaccination prediction error table in Table 10, we can see that:

Firstly, the overall prediction ability in the sample is good. The RMSE and MAE of all three dependent variables in the in-sample prediction are at a low level. Among them, the Y3 model has the best in-sample prediction accuracy, with RMSE and MAE of 0.032 and 0.025, respectively. The second is model Y2, with RMSE and MAE of 0.037 and 0.03, respectively. Finally, model Y1 has RMSE and MAE of 0.049 and 0.04, respectively. The average RMSE and MAE of the three models in the sample prediction are 0.04 and 0.03, respectively.

Secondly, regarding the out-of-sample prediction results: the best out-of-sample prediction performance is model Y2, with RMSE and MAE of 0.039 and 0.036, respectively. This data is the smallest among the three models, proving that its error is low and the model can better fit the actual situation. The models ranked second and third in performance are models Y3 and Y1, respectively. The average RMSE and MAE of the three models for out-of-sample predictions are 0.05 and 0.04, respectively.

Thirdly, the prediction accuracy is significantly improved compared with existing research literature. In the current similar research results:

To begin with, the prediction fitting constant index R^2^ is summarized. The model used to predict the demand for the vaccine in the Latur Government Medical College in Maharashtra, India, is the Winters additive model and the Simple seasonal model. The fitting constant R^2^ of the prediction results is 0.655 and 0.780, respectively [14]. For the Portuguese adult survey sample data, three models, multiple linear regression, extended regression, and an artificial neural network, are used to predict vaccine attention. The calculated R² are 0.56, 0.65, and 0.69, respectively [15].

In addition, the prediction error index RMSE is summarized. For the OWID data, the vaccination of Canada, France, Italy, and Israel is predicted. Among them, Canada has the best prediction accuracy, with an RMSE value of 0.1351 [11]. Based on the official vaccination data from the Statens Serum Institut (State Serum Institute) in Denmark, AR, ARIMA, Holt-Winters (clinical), and linear regression + LASSO, bagging, and weighted majority (WM) (network) models were used to predict vaccination, with an RMSE of 12.87 [13]. Based on the Italian government GitHub database, an ARIMA model was used to predict vaccination, with an RMSE of 9.83 [17]. Based on a public database (the specific platform was not specified, but it was an official/open vaccine dataset), four models, ARIMA, LSTM, Prophet, and Hybrid Harvest (ARIMA+Prophet), were used to predict vaccination, with an RMSE of 0.3049 for the optimal model [19].

Finally, the prediction error indicator, MAE, was summarized. Based on the Italian government GitHub database, an ARIMA model was used to predict vaccination, with an MAE of 7.35 [17]. Based on a public database (the specific platform was not specified, but it was an official/open vaccine dataset), four models, ARIMA, LSTM, Prophet, and Hybrid Harvest (ARIMA+Prophet), were used to predict vaccination, with the MAE of the optimal model being 1.42 [19].

These values were directly extracted from the results reported in the cited references, rather than recalculated from raw data.

The average fitting coefficient R^2^ of this paper is 0.97, the average RMSE and MAE of the in-sample predictions are 0.04 and 0.03, and the average RMSE and MAE of the out-of-sample predictions are 0.05 and 0.04. The higher the model fitting coefficient, the better. The fitting coefficient of this paper is far superior to the existing similar research results. The smaller the RMSE (root mean square error) and MAE (mean absolute error), the better.

The RMSE and MAE obtained in this paper are much smaller than those reported in existing studies [11,13,17,19], indicating that the ARDL model constructed in this paper has good prediction accuracy. Previous studies have used heterogeneous datasets, such as OWID African vaccination data [10], COVID-19 case growth, ICU admissions, mortality, and vaccination coverage in Canada, France, Italy, and Israel [11], U.S. questionnaire data [12], the official vaccination registry in Denmark [13], survey samples from Portugal [15], Romanian government data [16], Italian government GitHub data [17], and others [14,18,19]. Some of these studies did not report error metrics such as R², RMSE, or MAE due to differences in modeling approaches [12,16,18]. Nevertheless, when comparable error indicators were available [11,13-15,17,19], the ARDL model constructed in this paper consistently demonstrated relatively higher prediction accuracy. Our model achieved high fit and competitive out-of-sample accuracy within our dataset.

Wavelet coherence analysis

Although the ARDL model used above reveals the existence of a long-term cointegration relationship between vaccination and search index, and the test has passed the argument of the cointegration equation, it cannot capture the dynamic synergy characteristics between vaccination and information search behavior at different times and frequencies, which are very important for the correct formulation of policy interventions. Therefore, this paper further introduces wavelet coherence analysis to study the correlation and phase relationship between search index and vaccination behavior in the time-frequency domain, so as to identify the impact of short-term disturbances and high-frequency responses.

Through theoretical analysis, it is known that wavelet coherence technology can simultaneously capture the causal relationship and correlation between two variables. It combines information in the time domain and frequency domain and can mine potential undiscovered information between two time series [31]. All the wavelet coherence graphs below, Y1, Y2, and Y3, represent the wavelet coherence graphs between the US weekly vaccinations, weekly people vaccinated, weekly people fully vaccinated, and Google Trends search popularity. This figure shows the correlation and phase relationship between the two time series in the time-frequency domain.

This paper uses programming to perform wavelet coherence analysis. Specifically, a MATLAB program is used to generate wavelet coherence plots. The wavelet coherence was computed using MATLAB’s wcoherence function with the default analytic Morlet wavelet. Key parameters were set to VoicesPerOctave = 12, NumScalesToSmooth = 12, and PhaseDisplayThreshold = 0.5, with frequency limits adjusted to the data sampling interval.

Interpretation of the Wavelet Coherence Diagram

Coordinate axis: The horizontal axis is Date, which represents time, and the time range is from mid-December 2020 to early April 2022. This period covers the peak period of COVID-19 vaccine promotion in the United States and the subsequent stage. The vertical axis is the Y-axis Frequency (Hz). The higher the frequency (upward), the shorter the cycle (faster fluctuations), and the lower the frequency (downward), the longer the cycle.

Color: Indicates the correlation strength between Y_i_ and GT at this time and frequency. Yellow or orange areas indicate high coherence (close to 1), indicating that Y and GT change highly synchronously. The blue area indicates low coherence (close to 0), indicating that the relationship between the two is not obvious.

Arrow: Indicates the phase relationship, indicating the phase lag or lead relationship between GT (Google Trends) and Y. The arrow points to the right (→), which indicates a synchronous relationship (i.e., in phase). The arrow points up (↑) indicates that Y leads GT (GT lags). The arrow points down (↓) indicates that GT leads Y (Y lags). The arrow points to the left (←) indicates anti-phase, that is, the trend of the variable change is opposite. The arrow is diagonal (↗ ↘ ↙ ↖), indicating that there is both a time lag and an intensity difference between the two.

White dashed line: The area outside the white dashed line in the wavelet map represents the area with a large boundary effect, and the analysis results are unstable. Usually, research only focuses on the area within the dashed line.

Wavelet Coherence Analysis Between Y1 and GT

**Figure 5:**
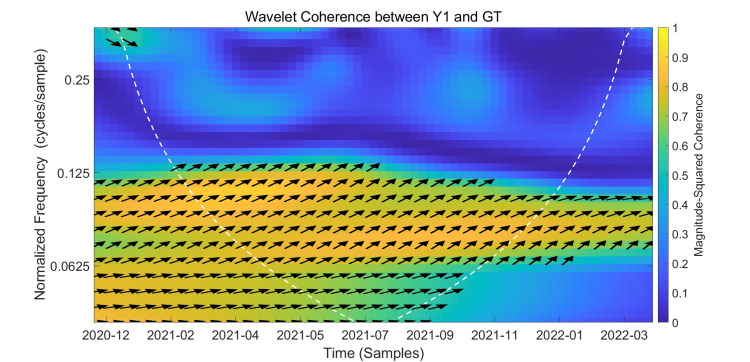
Wavelet Coherence Between Y1 and Google Trends The x-axis represents the timeline from December 2020 to February 2022. The y-axis denotes frequency in Hertz (Hz), with lower frequencies indicating long-term relationships and higher frequencies corresponding to short-term dynamics. Color intensity reflects the magnitude-squared coherence between Y1 and Google Trends. The black arrows indicate phase relationships, and the white dashed curve outlines the cone of influence. This figure illustrates the wavelet coherence analysis between Y1 (weekly vaccinations) and Google Trends search interest. The color intensity represents the strength of coherence in the time-frequency domain. Warmer colors (e.g., yellow) indicate higher coherence. The white contour lines denote areas of statistical significance at the 5% level.

The results of the wavelet coherence analysis between Y1 and Google Trends are presented in Figure 5, which shows the wavelet coherence plot of the Y1 and GT variables. The following analyzes the relationship between Y1 and GT based on this plot. Based on wavelet analysis theory, this article only discusses the reliable region within the white dashed "cone of influence."

First, we will discuss the strong coherence between the two variables. The correlation coefficient in this strong coherence region is approximately 0.6-1. The figure shows that between February 2021 and November 2021, a continuous yellow band of high coherence appeared in the frequency range of 0.06-0.13 Hz (approximately 8-16 cycles). The arrow in this region, to the upper right of the arrow, indicates that Y1 and GT are primarily synchronized, indicating a positive correlation between the two variables, with GT leading Y1. From a phase perspective, GT leads Y1 by approximately 1-3 weeks. At the lower frequency range of 0.04-0.06 Hz, a warm region was also observed in mid-2021, with arrows still primarily denoted by ↗, indicating that GT still leads Y1.

Second, we will discuss the weak coherence region. Weakly coherent regions are colored blue or cyan, with correlation coefficients ranging from 0 to 0.4. As can be seen in the figure, high frequencies ≥0.18 Hz are mostly blue, indicating weak synchronization between Y1 and GT. In this region, the correlation between the two variables cannot be discerned, indicating a very weak correlation. The right side of the figure also reveals a significant weakening or instability in the coherence between the two variables, also indicating a very low correlation coefficient in this region.

In summary, the wavelet coherence plot of Y1 and GT shows that the coherence between Y1 and GT is strongest in the medium- to long-term period of 2021 and then weakens, reflecting the phased nature of the correlation between the two variables. From a phase perspective, the two are positively correlated, with GT leading Y1 by approximately 1-3 weeks. This supports a causal relationship between search and vaccination, which can be used for preemptive monitoring and forecasting of short- and medium-term vaccination demand.

Wavelet Coherence Analysis Between Y2 and GT

**Figure 6:**
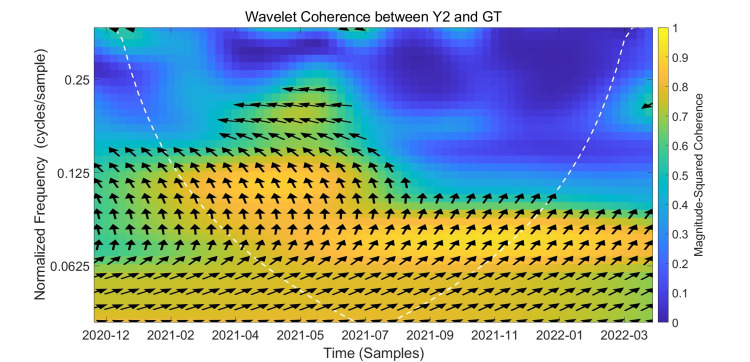
Wavelet Coherence Between Y2 and Google Trends The x-axis represents the timeline from December 2020 to February 2022. The y-axis denotes frequency in Hertz (Hz), with lower frequencies indicating long-term relationships and higher frequencies corresponding to short-term dynamics. Color intensity reflects the magnitude-squared coherence between Y2 and Google Trends. The black arrows indicate phase relationships, and the white dashed curve outlines the cone of influence.This figure shows the wavelet coherence between Y2 (weekly people vaccinated) and Google Trends  search interest over time and frequency. The color scale reflects the magnitude of coherence, with warmer colors indicating stronger correlation. Statistically significant areas at the 5% level are outlined by the white contour lines.

 The results of the wavelet coherence analysis between Y2 and Google Trends are presented in Figure 6. First, let's examine the strong coherence between the two variables in Figure 6. The figure shows a continuous yellow-orange band of high coherence between the bottom and mid-low frequencies in the time-frequency range of 0.06-0.13 Hz from February 2021 to October 2021, with arrows mostly pointing to the upper right. The direction of the arrows indicates that Y2 and GT move in the same direction, demonstrating a positive correlation between them. GT leads Y2 by approximately 1-3 weeks. In addition to the yellow region at the bottom of the figure, a triangular warm region appears between 0.15-0.22 Hz from April 2021 to July 2021. However, the high coherence is shorter-lived, and the arrows in this region are also primarily marked with ↗, indicating that GT also exhibits leading characteristics in this short-period fluctuation.

Next, we discuss the weak correlation region. The figure shows that in the high-frequency region ≥ 0.25 Hz, the image color is predominantly blue-green, indicating weak synchronization and poor correlation between the two. In summary, wavelet coherence analysis shows that Y2 (the number of new cases receiving at least one dose) and Google Trends have the strongest correlation in the 8-16 week period in 2021. In the temporal relationship, the dominant phase indicates a positive correlation between the two, with GT leading Y2, indicating that GT can be used as a predictor of Y2.

Wavelet Coherence Analysis Between Y3 and GT

**Figure 7:**
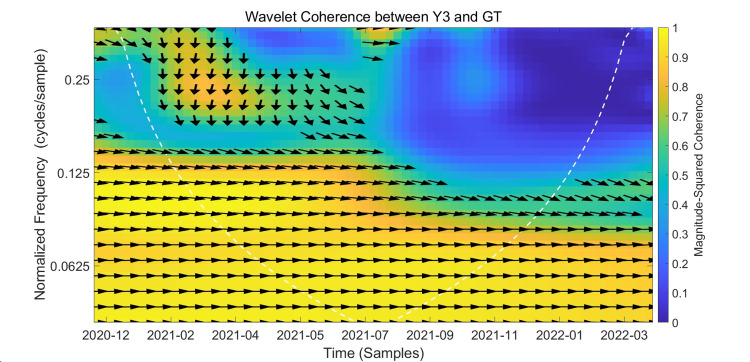
Wavelet Coherence Between Y3 and Google Trends The x-axis represents the timeline from December 2020 to February 2022. The y-axis denotes frequency in Hertz (Hz), with lower frequencies indicating long-term relationships and higher frequencies corresponding to short-term dynamics. Color intensity reflects the magnitude-squared coherence between Y3 and Google Trends. The black arrows indicate phase relationships, and the white dashed curve outlines the cone of influence. This figure shows the wavelet coherence between Y3 (weekly people fully vaccinated) and Google Trends search interest over time and frequency. The color scale reflects the magnitude of coherence, with warmer colors indicating stronger correlation. Statistically significant areas at the 5% level are outlined by the white contour lines.

The results of the wavelet coherence analysis between Y3 and Google Trends are presented in Figure 7, which shows the wavelet coherence plot of the Y3 and GT variables. Similarly, the following analysis will be divided into strong and weak correlation regions.

First, we will discuss the analysis of strong correlation regions. The figure shows three strong correlation regions. The first region, from December 2020 to March 2022, at 0.05-0.10 Hz, exhibits a broadband high-correlation region with arrows pointing almost exclusively to the right, indicating that Y3 and GT are synchronized; that is, a positive correlation exists between the two variables. GT also exhibits a slight lead (time range <1 week). The second region, from February 2021 to August 2021, at 0.1-0.125 Hz, is warm in color and has arrows pointing mostly to the right or upper right, indicating a positive correlation between the variables, with GT leading Y3 by approximately 1-2 weeks. The third region, from January 2021 to April 2021, appears in the upper left corner within the 0.22-0.30 Hz period. Arrows pointing downward or left within this region can be interpreted as indicating completion of full vaccination and subsequent search interest.

Next, we analyze weakly correlated regions. Weakly correlated regions, colored blue or cyan in the figure, occur at frequencies ≥ 0.18 Hz. The correlation between variables in this region is weak, failing to capture the underlying relationship. Another weakly correlated region, colored blue, occurs within the medium- and high-frequency range from October 2021 to May 2022, indicating very weak correlation and uncertain relationships between variables.

In summary, Y3 (weekly increments in completed vaccinations) exhibits the strongest and most persistent correlation with Google Trends at medium and low frequencies, stronger than at high and short-term periods. Time series analysis reveals a similar leading relationship between GT and Y3, with a lead of approximately 1-2 weeks, suggesting that GT has predictive properties for Y3.

Summary of Wavelet Coherence Analysis

Based on the wavelet coherence analysis of Y1, Y2, Y3, and GT, the following conclusions can be drawn:

GT has a predictive effect on vaccination behavior. As can be seen from the above, GT has a leading function on Y, but the leading time for Y1, Y2, and Y3 is different. Because GT is a search index that reflects the public's attention to vaccination and risk perception, the empirical analysis also shows that for each Y variable, the empirical results show a clear "search-action" path, that is, the public may actually perform vaccination behavior after information search.

The strong coherence areas between GT and vaccination behavior are different. Y3 has the strongest coherence and the longest duration, forming a clear orange-yellow continuous band in the 10-20 week period. Y2 has the second strongest coherence, showing a continuous warm color in the 8-16 week period. The arrows in multiple time periods show that GT stably leads Y2. Y1 has a medium coherence, and a high coherence area appears in the 8-16 week period.

Wavelet coherence analysis can reveal dynamic synergistic characteristics at different frequencies. The long-term cointegration relationship revealed by the ARDL model, and the results of wavelet coherence analysis show that the strong coherence of Y1, Y2, Y3 and GT is mainly concentrated in the core time-frequency band of 0.05-0.16 Hz (period of about 6-20 weeks) from February to November 2021, reflecting the phased linkage and frequency dependence characteristics of "search popularity" and vaccination behavior during the peak of the epidemic. This conclusion shows that the vaccination behavior response to the network search index is more sensitive in the early stage of the epidemic, and the reason for the weakening of the response in the later stage may be due to information weakening or policy influence. The policy implication of this research conclusion is that when responding to major infectious disease epidemics, different response measures must be taken according to the different stages of the epidemic, such as using a search index for prediction in the early stage, and combining policy factors for comprehensive consideration in the later stage.

Policy implications

Based on the above conclusions, a "search popularity-vaccination action" linkage monitoring mechanism can be constructed, and a vaccination early warning system based on Google Trends can be established. By monitoring the popularity of keyword searches in real time, when there is an increase for several consecutive weeks, it should be regarded as a harbinger of a surge in willingness to be vaccinated, so that corresponding resources can be deployed 1-3 weeks in advance, ultimately achieving the effect of efficiently responding to changes in the epidemic.

## Discussion

Key differences of present study to existing literature 

This study differs in several key aspects compared with existing studies [7,13,17,19]. First, we used a highly interpretable autoregressive distributed lag model instead of a black-box machine learning model, making it more suitable for real-world policy simulations. Second, we verified not only the predictive power of the search index but also its long-term cointegration relationship with vaccination behavior, which is often overlooked in previous studies [7,16]. Third, the introduction of wavelet coherence analysis provides new insights into the time-frequency heterogeneity of this relationship and reveals the lag effect, especially in the context of widespread vaccination. Finally, our model shows excellent predictive performance both in-sample and out-of-sample, highlighting its potential as a tool for public health forecasting and decision-making.

Significance of the ARDL model in the improvement of vaccine prediction

The ARDL model can significantly improve the accuracy of vaccination prediction. Existing research results show that the search index (Google Trends) has a predictive function [17]. Based on existing research, this study can significantly improve the prediction accuracy by introducing the ARDL model [28]. The empirical calculation results of this paper show that the relevant prediction indicators of the ARDL model: the average value of the fitting coefficient R^2^ is 0.97, the average value of the RMSE is 0.05, and the average value of the MAE is 0.04. The average accuracy of existing studies: R^2^ is about 0.75, RMSE is about 2.7, and MAE is about 3.19. Although the data used in this paper is different from the data of other research results, it still has a certain reference and comparison value. The reason for the improvement in accuracy is mainly that the ARDL model has the ability to automatically confirm the optimal lag order according to AIC, thereby significantly improving the prediction accuracy.

Significance of wavelet coherence analysis in vaccine prediction

Wavelet coherence analysis has practical guiding value in vaccination prediction. The conclusion of wavelet coherence analysis shows whether Y1, Y2, and Y3 are synchronized in different time periods and frequency ranges, thereby helping the public understand the response mechanism of search behavior to vaccination behavior and providing practical support for the formulation of multi-stage prevention and control measures. In the current research prospect of global fitting based on linear or deep learning, this paper proposes to use wavelet coherence as a research tool to provide a more explanatory method from the perspective of time and frequency, and provide a new research paradigm for introducing frequency domain analysis into traditional time domain prediction in the future, thereby enriching the quantitative analysis theory of public network search behavior and infectious disease vaccination behavior.

Establishment of a “search-vaccination” monitoring mechanism to perceive vaccine demand trends in advance

According to the vaccination prediction model based on search index proposed in this paper, public health management departments can use search index as a substitute variable for public network information behavior, identify changes in public attention, and capture the demand for vaccines, so as to prepare for vaccine demand in advance. By building a search early warning mechanism, they can prepare for material allocation in advance, and ultimately achieve the goal of increasing vaccination rates.The operational steps of the “Search for Vaccines” monitoring mechanism can be divided into the following steps: (1) Search keyword setting and data acquisition. (2) Data processing and index construction. (3) Warning threshold setting. This part can be determined based on historical experience and expert advice. (4) Real-time monitoring and model prediction. (5) Multi-department coordinated response. (6) Evaluation and optimization.

Strengthen behavioral guidance and policy response for fully vaccinated groups

From the research conclusions of this paper, it can be seen that Y3, that is, fully vaccinated behavior, has a delayed response to the search index. It is recommended to strengthen policy guidance and increase publicity efforts during vaccination (i.e., the second shot or booster shot), and encourage the public to recognize the importance of vaccination through various channels, so as to make up for the delayed effect of search information response.

Promote the construction of an intelligent prediction system based on multi-source data fusion

The model constructed in this paper is an ARDL model with a single independent variable, and the data source is too single. It is suggested to integrate multiple network information in practical applications, such as Weibo data, news data, etc., and build a more complex and more accurate vaccine prediction system by integrating multi-source data, so as to improve the prediction accuracy and achieve the unity of real-time, accuracy and adaptability. 

Understanding vaccination behavior in the context of COVID-19 sequelae

Through the empirical research of this paper, it was found that there is a significant correlation between online search behavior and vaccination rate. This research result can not only reflect the impact of public information search behavior on public health practice, but also the conclusion can be applied to the analysis and understanding of other health behaviors. As mentioned in references [32][33], COVID-19 sequelae may have a profound impact on individual health behaviors, and existing studies on the evaluation of post-COVID syndrome have shown that chronic or complicated symptoms after viral infection will strengthen the public's attitude towards health risks and promote the public to adopt more positive health behaviors. In this case, through the Google search index, we can timely understand the public's concerns about long-term complications after infection and predict the public's future prediction motivation. By combining this study, we can understand the coupling relationship between search behavior and vaccination behavior from a new perspective and provide reference data for existing theories.

Significance of the study in the context of existing literature

This section explains the importance of this study. Although existing literature has noted the monitoring potential of search data in vaccination prediction [7, 13, 17]. For example, using Google Internet search data and Italian vaccination dose data, autoregressive integrated moving average (ARIMA) models were used to predict vaccination data [17]. However, most studies currently use qualitative or a small amount of quantitative analysis methods to study the relationship between search data and actual participation in vaccination behavior [13, 17]. For example, official vaccination clinical data were combined with Google Trends data to predict vaccination. The research dataset is clinical vaccination data with a small amount of data [13]. Current research has the problem of low quantitative prediction accuracy [11, 13, 17]. For example, using the data from OWID, a regression ARIMA model was used to predict vaccination coverage in Canada, France, Italy, and Israel. The highest prediction accuracy was for Canada, with an error index RMSE of 0.1351. The error accuracy is low [11]. This paper introduces the ARDL model and combines it with the wavelet coherence analysis method to achieve the purpose of significantly improving prediction accuracy and capturing time-frequency heterogeneity. For example, the average fitting coefficient R^2^ of this paper is 0.97, the average RMSE and MAE of the in-sample predictions are 0.04 and 0.03, and the average RMSE and MAE of the out-of-sample predictions are 0.05 and 0.04. These index values ​​are all better than existing research results. The research method of this paper provides a more comprehensive research framework. Compared with the published research results [10,11,13,17], this study advances the methodological tools of digital epidemiology, combines digital information with advanced models, and improves the prediction capabilities. For example, the use of mixed time series models is compared to predict the vaccination rate of COVID-19 in Africa. However, this study did not involve frequency analysis [10]. Another example, the error indicators R^2^, RMSE, and MAE of the in-sample prediction of weekly people fully vaccinated (Y3) in this study are 0.98, 0.032, and 0.025, respectively. These values ​​are advantageous compared with existing research results [11,13,17]. Therefore, it can be considered that the model proposed in this paper provides a new method for predicting vaccination rates and comparing frequency domain heterogeneity. The research conclusions of this paper can provide objective reference data for real-time monitoring and decision-making of public health.

Research limitations

Discussion on the limitations of the study with a single influencing factor. This study only selected the Google Trends search index as the core independent variable to explore its relationship with vaccination. One of the limitations of this study is that the control variable is single, and other control variables are not integrated. We realize that the failure to include more control variables (such as prevention and control measures taken by the government, socio-political and economic variables, etc.) may affect the external validity of the research results. The main reason is that the goal of this study is methodological exploration, aiming to demonstrate the application potential of ARDL and wavelet coherence analysis in capturing the dynamic relationship between search behavior and vaccination behavior, so a relatively simple variable setting is adopted. However, future research can further integrate epidemic-related indicators (such as the number of new cases, mortality rate), policy variables (such as government prevention and control index, compulsory vaccination measures), and socioeconomic factors, so as to more comprehensively study the impact of multiple control variables on vaccination, and provide more complete reference data for better epidemic prevention and control.

The second limitation is that the prediction model used in this paper is a linear ARDL model, which fails to consider possible nonlinear problems. Although the conclusions of this study are explanatory and the prediction accuracy is high, it does not take into account the possible nonlinear problems between variables, so it may not be able to more accurately simulate the nonlinear dynamic process caused by the adjustment of vaccination policies.

Although this study has the above limitations, the prediction model and frequency domain analysis method proposed in this paper are still a new attempt, thus providing a theoretical basis for more in-depth research in the future.

## Conclusions

This paper examines the relationship between search index and vaccination behavior from both a temporal and frequency perspective, providing a new research paradigm for studying the relationship between public health attentions and health behaviors from multiple perspectives.

This study comes to the following conclusions: First, constructing an ARDL model based on a search index improves the prediction accuracy of vaccination. Second, a long-term cointegration relationship between the search index and vaccination is established. Third, wavelet coherence analysis reveals time-frequency heterogeneity between the search index and vaccination.

This study's conclusions are valuable for public health policy-making, and its research methods and conclusions can provide a basis for the prevention and control of major infectious diseases that may recur in the future.
